# Effects of Chronic Inhalation of Electronic Cigarette Vapor Containing Nicotine on Neurotransmitters in the Frontal Cortex and Striatum of C57BL/6 Mice

**DOI:** 10.3389/fphar.2019.00885

**Published:** 2019-08-12

**Authors:** Fawaz Alasmari, Laura E. Crotty Alexander, Alaa M. Hammad, Christine M. Bojanowski, Alex Moshensky, Youssef Sari

**Affiliations:** ^1^Department of Pharmacology and Experimental Therapeutics, College of Pharmacy and Pharmaceutical Sciences, University of Toledo, Toledo, OH, United States; ^2^Department of Pharmacology and Toxicology, College of Pharmacy, King Saud University, Riyadh, Saudi Arabia; ^3^Pulmonary and Critical Care Section, VA San Diego Healthcare System, San Diego, CA, United States; ^4^Department of Medicine, Division of Pulmonary and Critical Care, University of California at San Diego (UCSD), La Jolla, CA, United States; ^5^Department of Pharmacy, College of Pharmacy, Al-Zaytoonah University of Jordan, Amman, Jordan

**Keywords:** E-cigarettes, dopamine, glutamate, glutamine, GABA, serotonin

## Abstract

Electronic (E)-cigarettes are the latest form of nicotine delivery device and are highly popular in the general population. It is currently unknown whether vaping E-cigarettes (E-CIGs) leads to nicotine addiction. Alterations in the levels of the neurotransmitters in the mesocorticolimbic areas have been reported to mediate the initiation and development of nicotine addiction. Therefore, to determine whether E-CIGs activate the same addiction pathways as conventional cigarettes, we investigated for the effects of daily inhalation of nicotine (24 mg/ml)-containing E-CIG vapor for 6 months on the concentrations of these neurotransmitters in the frontal cortex (FC) and striatum (STR) of male C57BL/6 mice as compared to control group that was exposed to air only. We reported here that 6-month E-CIG vapor containing nicotine inhalation decreased dopamine concentration only in the STR. There were no changes in serotonin concentrations in the FC or STR. Chronic E-CIG exposure also increased glutamate concentration in the STR alone, while glutamine concentrations were increased in both the FC and STR. We found that E-CIG exposure also decreased GABA concentration only in the FC. These data suggest that chronic E-CIG use alters homeostasis of several neurotransmitters in the mesocorticolimbic areas, which may result in the development of nicotine dependence in E-CIG users.

## Introduction

The use of electronic (E)-cigarettes worldwide is substantial, with use ranging from 1 to 25% across populations (Delnevo et al., 2015; Palipudi et al., 2015; Singh, 2016; Zhong et al., 2016). Although E-cigarette devices produce a nicotine-containing aerosol, commonly referred to as vapor, with fewer toxic constituents as compared to conventional tobacco cigarette smoke (Margham et al., 2016), research studies have reported that E-cigarette (E-CIG) vapor induces inflammation and impairs host defense and has other notable toxicological effects (Vardavas et al., 2012; Hwang et al., 2016; Yu et al., 2016; Canistro et al., 2017; Crotty Alexander et al., 2018). Indeed, 3–6 month exposure of nicotine (24 mg/ml)-containing E-CIG vapor in CD-1 and C57BL/6 mice through inhalation route resulted in an increase of circulating proinflammatory and profibrotic proteins in both strains. Moreover, chronic E-CIG vapor inhalation reduced renal filtration by 20%, and fibrosis was also shown in kidney, heart, and liver. Importantly, the same study found that E-CIG vapor inhalation altered the function of cardiovascular system; this involves reduction in the heart rate and increases in the blood pressure (Crotty Alexander et al., 2018). In addition, there is a high dependence rate reported for E-CIGs, and clinical studies have reported addictive behavioral effects such as withdrawal manifestations and a high urge to smoke (Dawkins et al., 2012; Foulds et al., 2014; Etter and Eissenberg, 2015). It has been suggested that these addictive effects develop due to alterations in the homeostasis of neurotransmitters in the mesocorticolimbic brain areas (Mifsud et al., 1989; Caillé and Parsons, 2004; Deehan et al., 2015). In our study, for the first time, we reported the effects of chronic inhalation of nicotine-containing E-CIG vapor on the tissue contents of several neurotransmitters in the frontal cortex (FC) and striatum (STR) in male C57BL/6 mice.

Stimulation of nicotinic acetylcholine receptors (nAChRs) after exposure to nicotine using minipumps for 2 weeks at dose of 0.125 mg/kg (s.c.) or microinjection of nicotine into the ventral tegmental area (VTA) caused changes in dopamine neurotransmission in male Sprague–Dawley rats and male Wistar rats, respectively (Fuxe et al., 1990; Tizabi et al., 2002). Additionally, it has been found that dopamine receptor antagonists attenuated self-administration of nicotine (0.03 mg/kg/infusion) in male Long–Evans rats while increases in extracellular dopamine concentrations in the nucleus accumbens (NAc) shell have been observed in male Sprague–Dawley rats with a preference to nicotine (Corrigall and Coen, 1991; Scherma et al., 2012). A recent clinical report showed that smoking tobacco cigarettes also affected dopamine biosynthesis in the mesocorticolimbic system in humans (Rademacher et al., 2016). Nicotine exposure has also been shown to induce effects of serotonin neurotransmission. Acute (2, 4, or 8 mg/kg, s.c.) and chronic (0.4 mg/kg, i.p. for 2 weeks) systemic injection of nicotine caused an increase of serotonin release in the frontocortical area in male Sprague–Dawley rats and STR in male Wistar rats, respectively (Ribeiro et al., 1993; Takahashi et al., 1998). Chronic exposure, not acute, to nicotine (0.7 mg/kg, s.c.) increases the re-uptake of serotonin in the prefrontal cortex (PFC) and hippocampus in male Sprague–Dawley rats (Awtry and Werling, 2003). Chronic nicotine (6 mg/kg/day) exposure using minipumps for 12 days also decreases the mRNA expression of serotonin transporters in the dorsal raphe in male Wistar rats, suggesting alterations in the concentrations of serotonin within the mesocorticolimbic system (Semba and Wakuta, 2008). It has been found that there is an association between gene polymorphism of serotonin transporters and smoking behaviors in humans (Ishikawa et al., 1999; Watanabe et al., 2011). Less is known about the effect of chronic E-CIG exposure on the dopaminergic and serotonin systems. Here, we investigated dopamine and serotonin levels in both the FC and STR of mice after 6 month inhalation of nicotine-containing E-CIG vapor.

A prior study from our laboratory demonstrated that 6-month inhalation of E-CIG vapor containing nicotine (24 mg/ ml) led to down-regulation of both cystine/glutamate antiporter (xCT) and glutamate transporter-1 (GLT-1) in the STR of outbred female CD1 mice (Alasmari et al., 2017). Decreases in the expression of these astro-glial glutamate transporters are associated with high concentrations of glutamate in the synapses (Han et al., 2008; Das et al., 2015). Moreover, our laboratory recently reported that increased GLT-1 expression in PFC and NAc is associated with reduced nicotine drinking (0.07 and 0.14 mg/ml) behavior in female alcohol preferring rats (Sari et al., 2016). Studies have shown that nicotine exposure through intra-cortical infusions (12 mM) in male Wistar rats or *in vitro* application in isolated nerve terminals stimulates α7-nAChR in presynaptic glutamatergic neurons and consequently increases the release of glutamate (Wang et al., 2006; Konradsson‐Geuken et al., 2009). The biosynthesis of glutamate and glutamine is also increased in animals exposed to nicotine (2mg/kg, s.c.) for 4 weeks in C57BL/6 mice (Shameem and Patel, 2012). In this study, we determined whether E-CIG vapor exposure causes alterations on the concentrations of glutamate and glutamine in the FC and STR.

Previous studies have reported that exposure to nicotine (10 μM) for short-term increased GABA neurotransmission in the medial septum and hippocampal CA1 pyramidal neurons (DuBois et al., 2013). GABA, an inhibitory neurotransmitter, blocks the effects of the excitatory neurotransmitter and dopamine in the brain (Dewey et al., 1999; Polosa and Benowitz, 2011), and GABA receptor agonists attenuate nicotine-seeking behaviors in male Wistar rats (Paterson et al., 2004; Paterson et al., 2005). Several studies have found that GABAergic system desensitization develops following long-lasting effects of nicotine (0.5 μM or 1 μM) using VTA neurons (Mansvelder et al., 2002; Pidoplichko et al., 2004). This desensitization effect reduces the inhibitory effects of GABA leading to excitability of the dopamine reward system (D’Souza and Markou, 2013). In this study, we evaluated the concentrations of GABA in the FC and STR of mice after long-term exposure to E-CIG vapors to investigate whether E-CIG use induces any alterations in the inhibitory neurotransmitter, GABA, and the excitatory neurotransmitters dopamine and glutamate.

## Materials and Methods

### E-CIG

E-liquid mixtures of 50% glycerin, 50% propylene glycol, and 24 mg/ml nicotine (purchased from Xtreme Vaping) were prepared in our laboratory as described in our recent study (Alasmari et al., 2017). No flavors or other additives were used. E-CIG cartomizers (tanks; 2.4 ohm, plastic, refillable) and batteries (rechargeable, stainless steel, 280 mAh fixed, automatic) were bought from FastTech. Fresh E-CIG vapors were created by activating the battery *via* application of negative pressure, 2L/ min for 1 s, by the InExpose system (SciReq), and followed by continuous negative pressure of 1L/min for 3 additional seconds. By activating the battery and applying pneumatic pressure, the e-liquid was heated and drawn through the internal atomizer, which created E-CIG vapors.

### Mouse Inhalation of E-CIG Vapor

Ten male C57BL/6 mice, 6–8 weeks old, were bought from Harlan Labs. The SciReq inExpose inhalation system was used as explained in our recent reports (Alasmari et al., 2017; Crotty Alexander et al., 2018). Soft-mesh restraints, such that only the noses of mice are introduced into the central channel through which the E-CIG vapor flows, were used in this study to focus the E-CIG vapor exposure to the respiratory system, creating a physiologic exposure model of E-CIG use. Mice inhaled E-CIG vapor for 4 s every 20 s, for 1 h/day, for 5 days/week, for 6 months (E-CIG group). Using the same restraints, control mice were exposed to environmental air only (air control group) for the same amount of time. Pre-warmed cages were used to recover mice for 30 min post-exposure. At the end of 6 months, mice were anesthetized with 10 mg/kg and 100 mg/kg of xylazine and ketamine, respectively, and were administered in PBS intraperitoneally, 30–60 min after the final exposure. Mice also received 200 units of heparin in 200 µl PBS i.p. during induction of anesthesia. Terminal intra-aortic bleed was performed, followed by opening the right and left cardiac ventricles. All animal protocols were in accordance with the NIH guidelines for animal use and approved by the IACUC committee at the University of California, San Diego, and San Diego VA Healthcare System.

### Brain Tissue Dissection

Brains were isolated and brain areas, FC and STR, were indicated and dissected using the Mouse Brain Atlas, the stereotaxic coordinates (Paxinos, 2004). All brain tissues were then snapfrozen in a liquid nitrogen and kept at −80°C for neurotransmitter assays.

### High-Performance Liquid Chromatography (HPLC) With Electrochemical Detection (EC)

The levels of dopamine and serotonin in the FC and the STR were detected using HPLC-EC system as described in our prior study (Das et al., 2016). Briefly, brain tissue samples were lysed and sonicated in 0.25N perchloric acid and subsequently centrifuged for 20 min at 4°C at 14,000 × g. A specific 0.22-μm filter was used to filter the supernatants, and the resulted filtrates were injected into a C18 column (3.2 × 150 mm, 3-μm particle size, from Thermo Scientific). A mobile phase (54.3 mM sodium phosphate, 0.32 mM citric acid, 0.215 mM octyl sodium sulfate, and 11% methanol [pH∼ 4.4]) was prepared and used. To detect dopamine and serotonin concentrations, the coulometric detector (CoulArray model 5600A, ESA, Inc). was applied and connected to CoulArray software in which the chromatograms are shown. The external standards of dopamine and serotonin were purchased from Sigma-Aldrich and analyzed by calculating the peak area representing the standard calibration curve. The concentrations of the dopamine and serotonin of the tissue samples were then detected. Tissue sample pellets were re-suspended with 1N NaOH for protein quantification, using DC (detergent compatible) protein assay, to normalize the concentrations of neurotransmitters to the relative protein contents.

HPLC-EC system was used to quantify the concentrations of glutamate, glutamine, and GABA in the FC and the STR following 6-month exposure to E-CIG vapor. As described previously (Das et al., 2016), homogenized FC and STR tissues with millipore water were heated at 98°C for 5 min and centrifuged for 5 min at 4°C at 10,000 rpm. After centrifugation, supernatants were filtered using 0.22-μm filters, while the pellets were collected for protein assay. Derivatization of pre-column (C18 column [3.0 × 50 mm, 2.5-μm particle size, Waters, Inc.]) of supernatants was performed by mixture of sodium sulfite and o-phthalaldehyde in solution-containing ethanol, sodium sulfite, and 0.1 M sodium tertraborate using 540 autosampler and ESA model. A mobile phase (0.1 mM EDTA, 0.1 M Na_2_HPO_4_, and 7.5% methanol [pH ∼ 2.8–3]) was prepared and used. To detect glutamate, glutamine, and GABA, the coulometric detector (CoulArray model 5600A, ESA, Inc). was applied and connected to CoulArray software in which the chromatograms are shown. The external standards of glutamate, glutamine, and GABA were purchased from Sigma-Aldrich and analyzed by calculating peak area which represent standard calibration curve. The concentrations of GABA, glutamate, and glutamine of the tissue samples were then detected. Tissue sample pellets were re-suspended with 1N NaOH for protein quantification, using DC (detergent compatible) protein assay, to normalize the concentrations of neurotransmitters to the relative protein contents.

### Statistical Analyses

Unpaired independent *t*-test was used to analyze tissue content readings obtained for neurotransmitters of interest in the FC and STR brain tissues between E-CIG-exposed group and air controls. The level of the significance of the statistical data was shown as *p* < 0.05.

## Results

### Chronic E-CIG Vapor Exposure for 6 Months Decreased Dopamine in the STR

**Figure 1:**
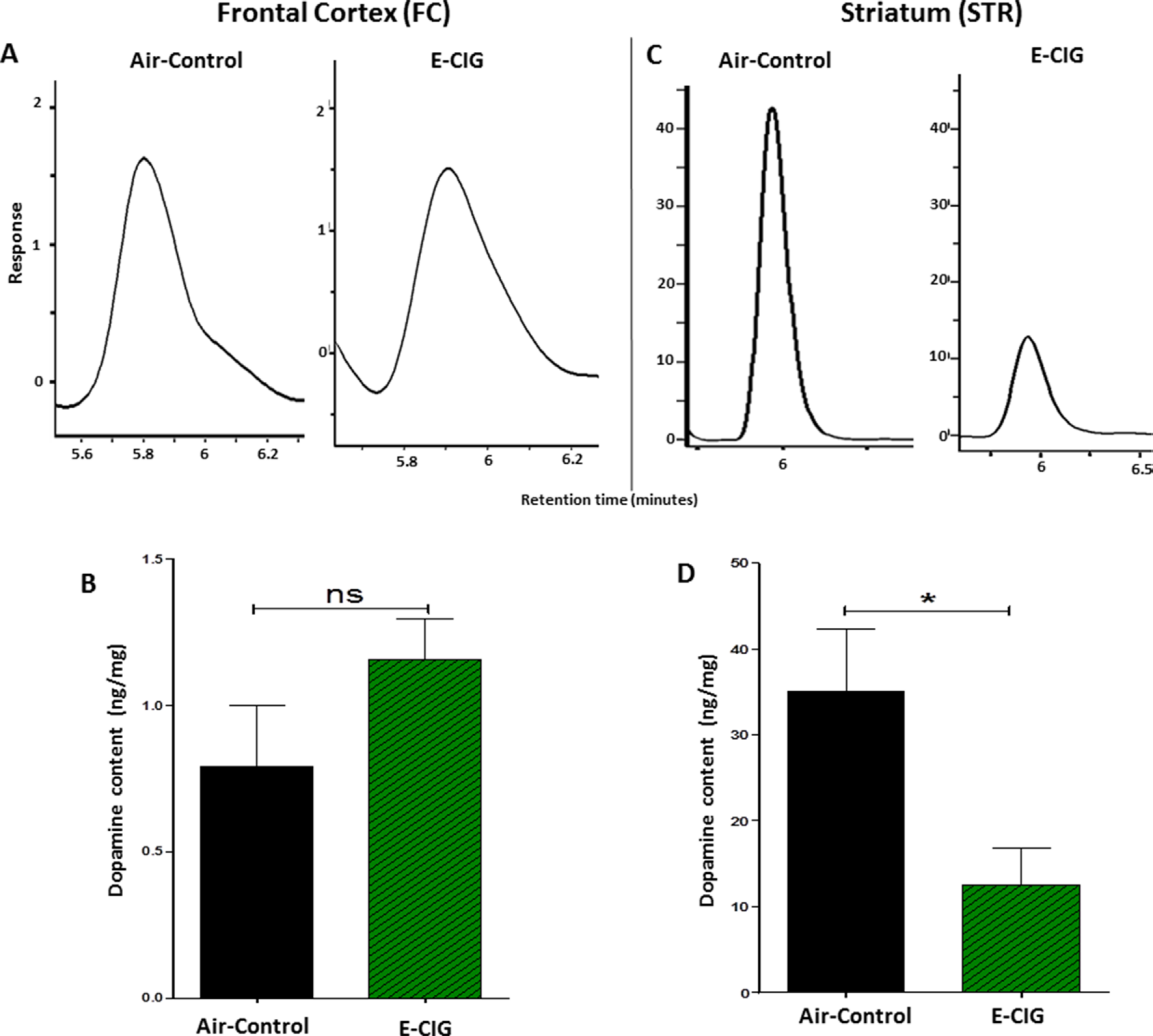
Effects of inhalation of E-cigarette vapor (E-CIG) for 6 months on dopamine concentrations in the frontal cortex (FC) and striatum (STR) in male C57BL/6 mice. **(A)** Peaks of dopamine in the FC in air and E-CIG groups. **(B)** Independent *t*-test analysis did not show any significant alterations in dopamine concentration in the FC between control and E-CIG groups. **(C)** Peaks of dopamine in the STR for air and E-CIG groups. **(D)** Independent *t*-test analysis revealed a significant reduction in dopamine concentration in the STR in E-CIG vapor–exposed group as compared to air controls. Data are reported as mean ± SEM (*p < 0.05, ns; not significant), (*n* = 5 for each group), (*X*-axis, retention time [minutes], *Y*-axis, response).

We determined the concentrations of dopamine in the FC and STR in mice exposed to only air- or nicotine-containing E-CIG vapor for 6 months. Compared to control mice, we demonstrated that the concentration of dopamine in the STR was lower in E-CIG group (*p* = 0.0266; **Figures 1C, D**). However, chronic E-CIG vapor exposure did not alter dopamine concentrations in the FC area (*p* = 0.1792; **Figures 1A, B**).

### Chronic E-CIG Vapor Exposure for 6 Months Does Not Alter Serotonin Concentrations in the FC or STR

**Figure 2:**
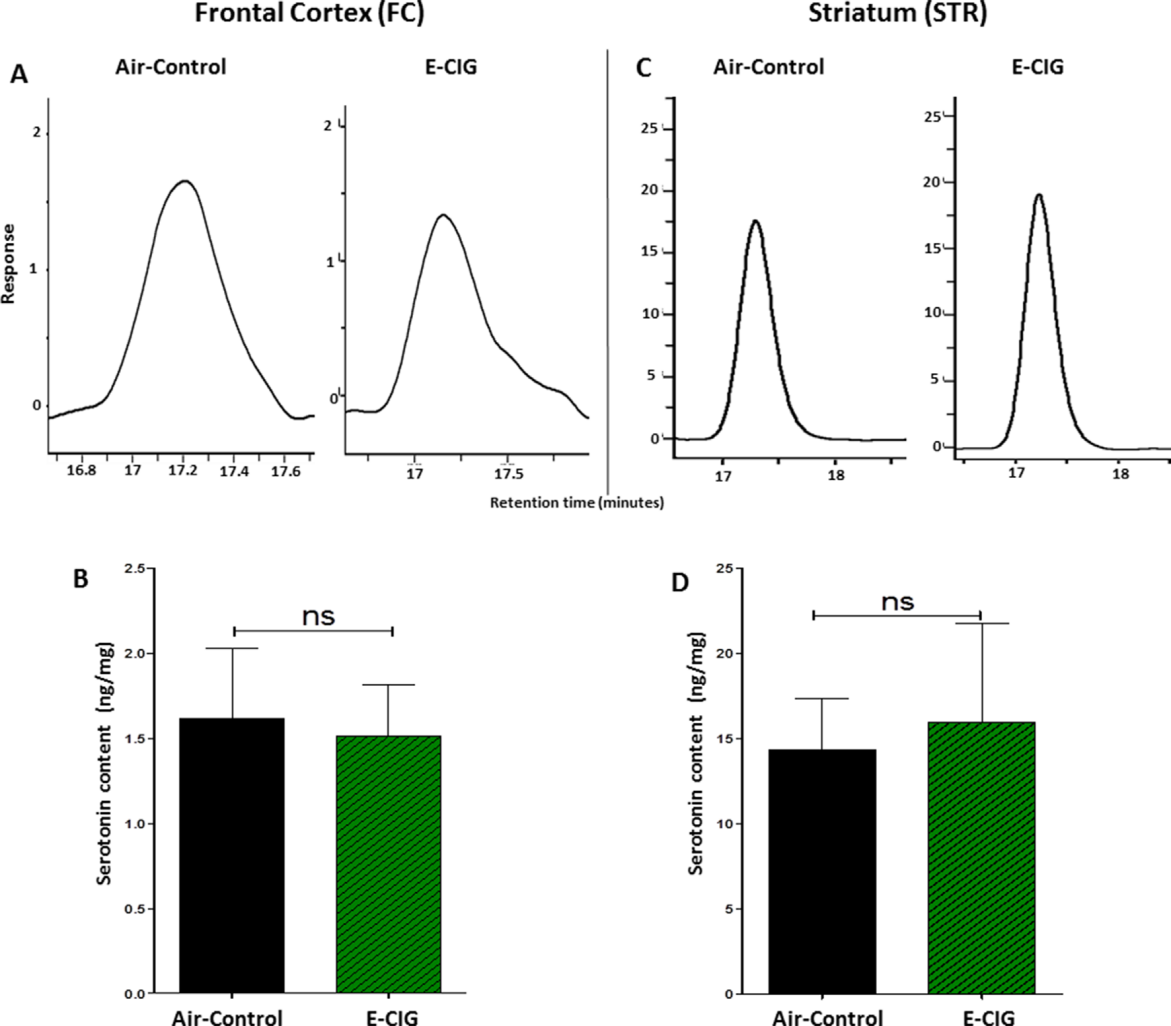
Effect of inhalation of E-CIG for 6 months on serotonin concentrations in the FC and STR in male C57BL/6 mice. **(A)** Peaks of serotonin in the FC in air controls and E-CIG groups. **(B)** Independent *t*-test analysis did not show any significant alterations in serotonin concentration in the FC between E-CIG and air controls. **(C)** Peaks of serotonin in the STR in air controls and E-CIG groups. **(D)** Independent *t*-test analysis did not reveal any significant alterations in serotonin concentration in the STR between E-CIG and air controls. Data are reported as mean ± SEM (ns, not significant), (*n* = 5 for each group), (*X*-axis, retention time [minutes], *Y*-axis, response).

We further detected the concentrations of serotonin in the FC and STR after inhalation of nicotine-containing E-CIG vapor for 6 months. There were no significant changes in serotonin concentrations in the FC (*p* = 0.8462; **Figures 2A, B**) and the STR (*p* = 0.8114; **Figures 2C, D**) after 6-month inhalation of E-CIG vapors as compared to air controls.

### Chronic E-CIG Vapor Exposure for 6-Months Increased Glutamate Concentrations in the STR

**Figure 3:**
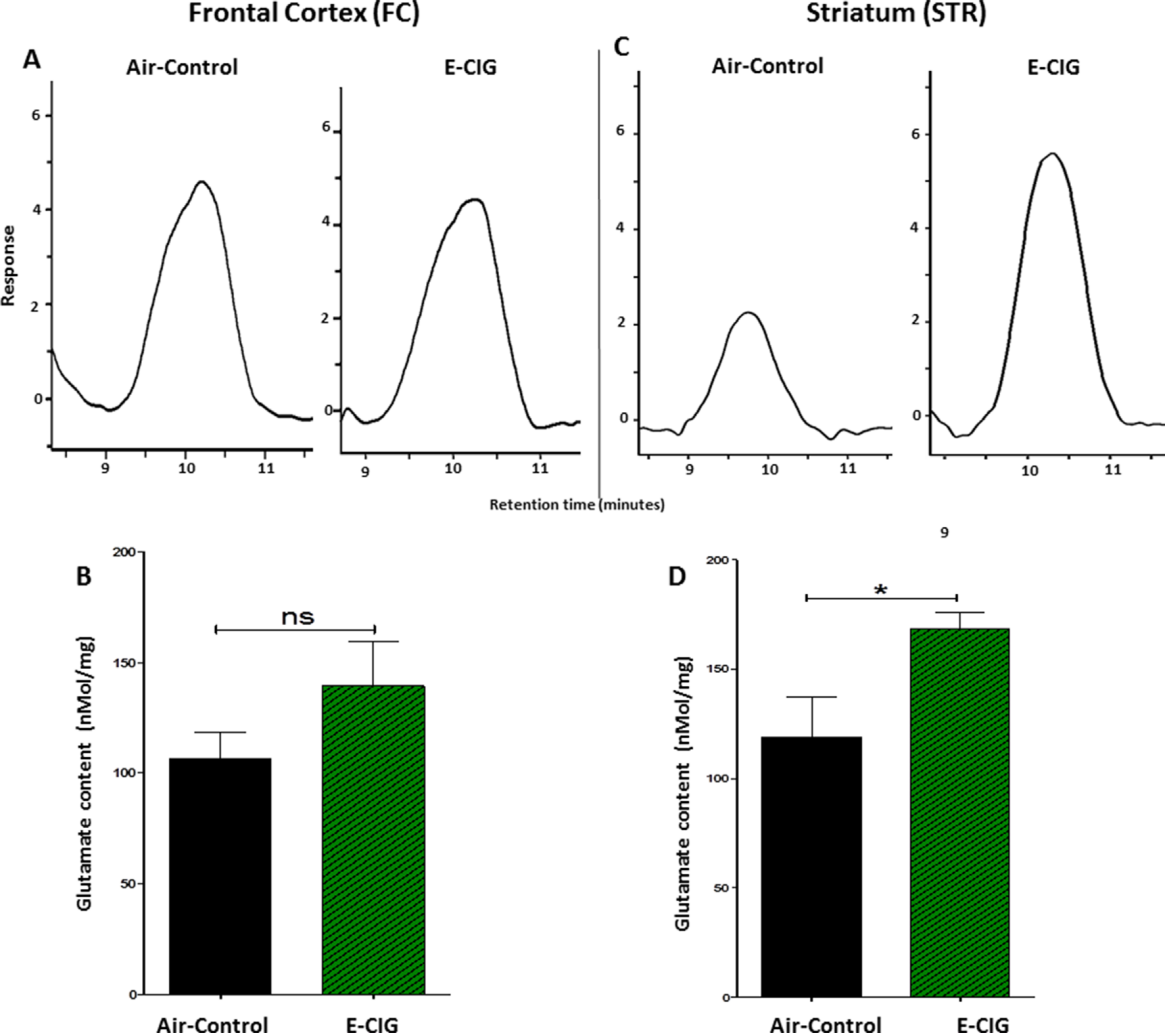
Effects of inhalation of E-CIG fox 6 months on glutamate concentrations in the FC and STR in male C57BL/6 mice. **(A)** Peaks of glutamate in the FC in air controls and E-CIG groups. **(B)** Independent *t*-test analysis did not show any significant alterations in glutamate concentration in the FC between E-CIG and air controls. **(C)** Peaks of glutamate in the STR for air control and E-CIG groups. **(D)** Independent *t*-test analysis revealed significant elevations in glutamate concentration in the STR in E-CIG vapor–exposed group compared to air controls. Data are reported as mean ± SEM (*p < 0.05, ns, not significant), (*n* = 5 for each group), (*X*-axis, retention time [minutes], *Y*-axis, response).

We quantified glutamate concentrations in the FC and STR in mice exposed to nicotine-containing E-CIG vapors for 6 months. As compared to air control mice, E-CIG-exposed group had a significant increase in glutamate concentration in the STR (*p* = 0.0366; **Figures 3C, D**) but not in the FC (*p* = 0.1995; **Figures 3A, B**). We next determined the effects of chronic exposure to E-CIG vapor on the concentrations of glutamine in both FC and STR.

### Chronic E-CIG Vapor Exposure for 6 Months Increased Glutamine Concentrations in the FC and STR

**Figure 4:**
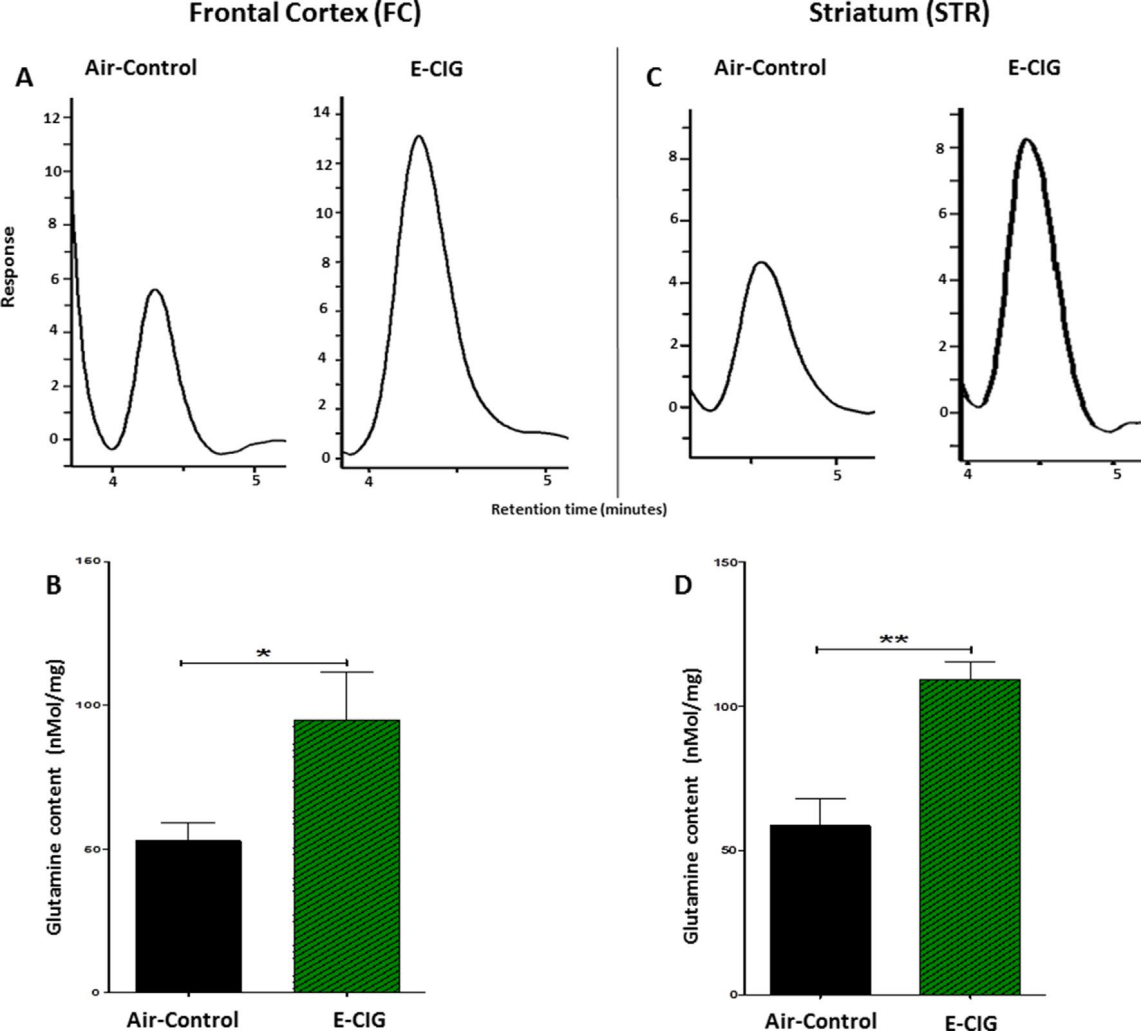
Effects of inhalation of E-CIG for 6 months on glutamine concentrations in the FC and STR in male C57BL/6 mice. **(A)** Peaks of glutamine in the FC in air control and E-CIG groups. **(B)** Independent *t*-test analysis revealed a marked increase in glutamine concentration in the FC in E-CIG vapor–exposed group as compared to the controls. **(C)** Peaks of glutamine in the STR in air control and E-CIG groups. **(D)** Independent *t*-test analysis revealed a marked increase in glutamine concentration in the STR in E-CIG vapor–exposed group compared to air controls. Data are reported as mean ± SEM (**p < 0.01,*p < 0.05) (*n* = 5 for each group), (*X*-axis, retention time [minutes], *Y*-axis, response).

Since daily exposure to E-CIG vapor for 6 months elevated glutamate concentrations in the STR, we evaluated concentrations of glutamine in both the FC and the STR in mice exposed to E-CIG vapor versus air controls for 6 months. Glutamine concentrations were significantly increased in the FC (*p* = 0.0481; **Figures 4A, B**) and STR (*p* = 0.0017; **Figures 4C, D**) in nicotine-containing E-CIG vapor–exposed group compared to air controls.

### Chronic E-CIG Vapor Exposure for 6 Months Decreased GABA in the FC

**Figure 5:**
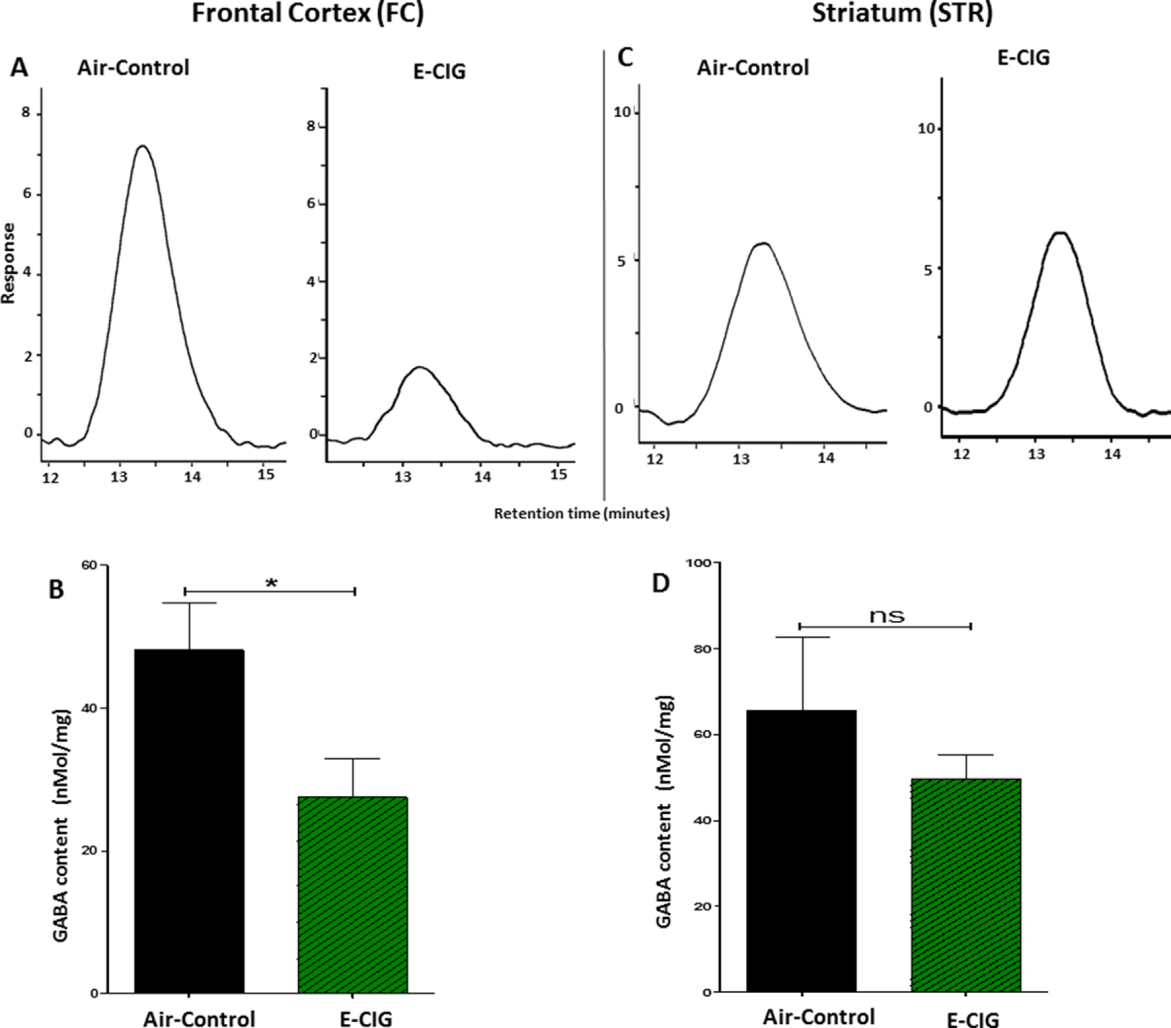
Effects of inhalation of E-CIG for 6 months on GABA concentrations in the FC and STR in male C57BL/6 mice. **(A)** Peaks of GABA in the FC in air control and E-CIG groups. **(B)** Independent *t*-test analysis revealed that there is a significant reduction in GABA concentration in the FC in E-CIG-exposed group compared to air controls. **(C)** Peaks of GABA in the STR in air control and E-CIG groups. **(D)** Independent *t*-test analysis did not show any significant alterations in GABA concentration in the STR between E-CIG-exposed group and air controls. Data are reported as mean ± SEM (*p < 0.05, ns, not significant), (*n* = 5 for each group), (*X*-axis, retention time [minutes], *Y*-axis, response).

The effects of 6 months inhalation of E-CIG vapors containing nicotine on the levels of GABA in the FC and STR were investigated. Chronic daily exposure to E-CIG vapor induced a significant decrease in the levels of GABA in the FC (*p* = 0.0415; **Figures 5A, B**) compared to air controls. However, there was no significant change in GABA concentration in the STR of mice after 6-month vaping of nicotine-containing E-CIG vapors (*p* = 0.3941; **Figures 5C, D**).

## Discussion

**Figure 6:**
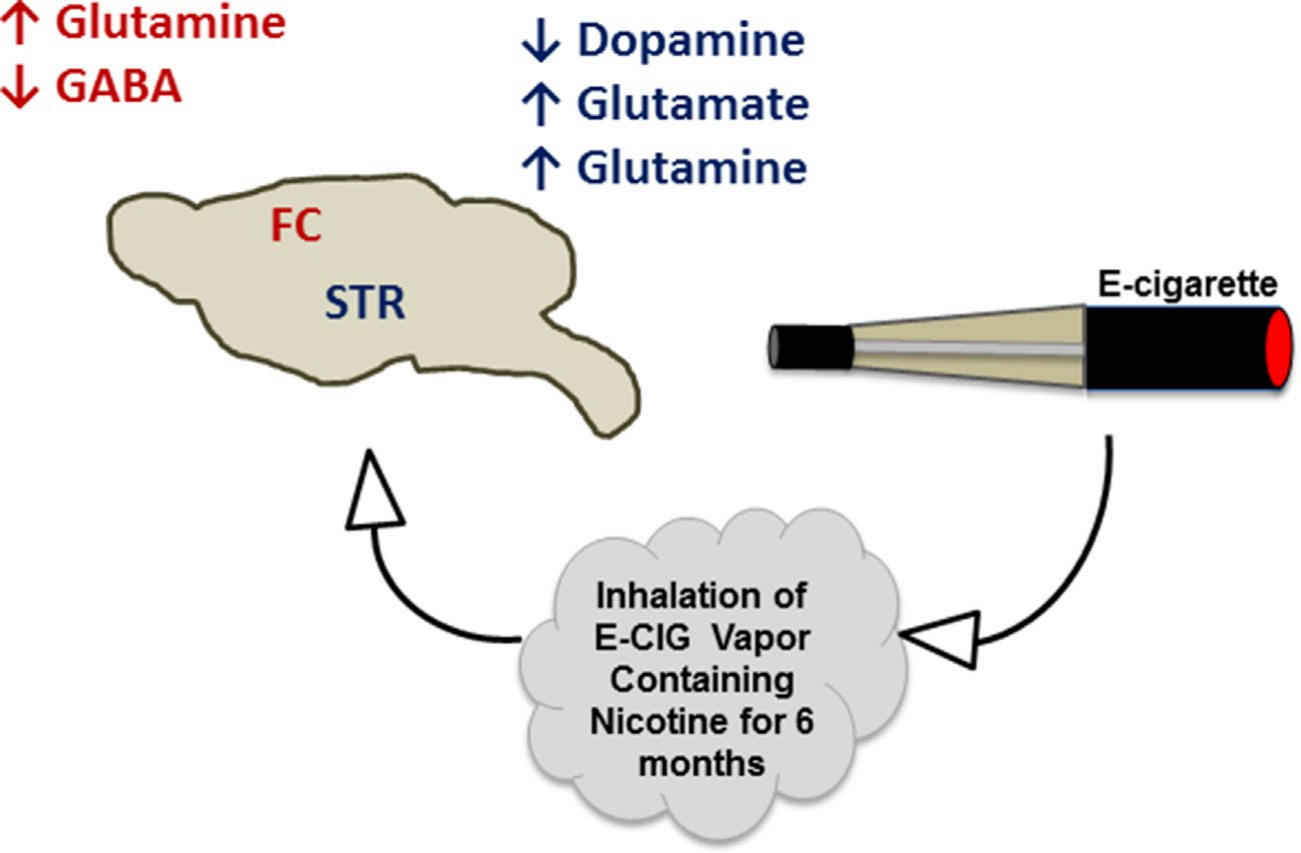
Schematic diagram summarizes the effects of inhalation of E-CIG vapor containing nicotine for 6 months on the concentrations of neurotransmitters in the FC and STR.

The projections of both glutamatergic and dopaminergic systems from the PFC and VTA, respectively, into the NAc, are necessary for the development of drug dependence, including nicotine (LaLumiere and Kalivas, 2008; Feduccia et al., 2012; Pistillo et al., 2015; Subramaniyan and Dani, 2015). Additionally, glutamatergic projections have been found to be released from the amygdala and hippocampus into the NAc (Goodwani et al., 2017; Alasmari et al., 2018). The FC receives GABAergic inputs from the VTA and basal ganglia (Carr and Sesack, 2000; Saunders et al., 2015), and GABA can inhibit dopamine release from the VTA into the NAc and FC. This effect has been suggested to attenuate nicotine- and cocaine-seeking behavior (D’Souza and Markou, 2013; Edwards et al., 2017). Alternatively, serotonergic inputs from raphe nuclei have been found to stimulate serotonin receptors in the STR, NAc, and hippocampus (Nakamura, 2013). In the present work, we studied the concentrations of these neurotransmitters in two critical brain areas, FC and STR, in mice exposed daily to nicotine within E-CIG vapor for 6 months. Our data provide evidence that chronic E-CIG use induces changes in neurochemical levels in the FC and STR and activates nicotine dependence pathways (**Figure 6**).

Nicotine exposure has been found to induce the release of neurotransmitters including dopamine and glutamate through stimulatory effects on nAChRs in the mesocorticolimbic areas (Tizabi et al., 2002; Tizabi et al., 2007; Konradsson‐Geuken et al., 2009; Yan et al., 2018). Exposure to nicotine (0.125 mg.kg, s.c.) using minipumps for 2 weeks increased dopamine stores in the caudate putamen in male Sprague–Dawley rats (Fuxe et al., 1990). This study found that the utilization of dopamine was reduced in the substantia nigra. Interestingly, other studies reported that several month-long exposure to nicotine through smoking cigarettes and drinking solution-containing nicotine significantly decreased dopamine synthesis and release in humans and monkeys, respectively (Perez et al., 2012; Rademacher et al., 2016). In this study, with a several month-long exposure to nicotine *via* E-CIG vapor inhalation, we found reduced dopamine concentrations in the STR, but not in the FC. This consistency between previous data and our own suggests that chronic inhalation of tobacco smoke or nicotine-containing E-CIG vapor reduces dopamine synthesis and content, specifically in the STR (**Figure 6**).

Previous studies have shown conflicting results regarding the effects of nicotine exposure on serotonin expression, uptake, and functions (Awtry and Werling, 2003; Munafo et al., 2005; Semba and Wakuta, 2008; Smolka et al., 2019). In male rats, acute nicotine (2, 4, or 8 mg/kg, s.c.) and chronic nicotine (0.4 mg/kg, i.p. for 2 weeks) exposure increased the extracellular concentrations and release of serotonin in the FC in Sprague–Dawley rats and STR in Wistar rats, respectively (Ribeiro et al., 1993; Takahashi et al., 1998). We did not find significant alterations in the concentrations of serotonin in the FC and STR following E-CIG vapor exposure for 6 months. These data suggest that, while nicotine might affect the release as well as the uptake of serotonin, there is no effect on the serotonin content within the FC and STR.

Studies found that glutamatergic system is dysregulated in multiple mesocorticolimbic brain regions following exposure to nicotine (Knackstedt et al., 2009; Cheng and Yakel, 2014). Using the same E-CIG procedure, we have reported changes in the expression level of xCT, GLT-1, α-7 nAChR, and GLAST in the STR, FC, and hippocampus in outbred female CD1 mice (Alasmari et al., 2017). Studies have reported that nicotine stimulates nAChRs and also reduces glutamate uptake, which may lead to elevation of synaptic glutamate concentrations (Knackstedt et al., 2009; Cheng and Yakel, 2014). In addition, studies showed that nicotine exposure (10 μM) increased glutamate transmission in hippocampal CA3 pyramidal neurons using patch-clamp recordings (Cheng and Yakel, 2014). Moreover, nicotine self-administration (0.03 mg/kg per infusion) for 21 days was found to cause downregulation of astro-glial glutamate transporters in the NAc in male Wistar rats (Knackstedt et al., 2009). This is suggested to decrease glutamate uptake and increase extracellular glutamate.

Importantly, we found recently that E-CIG vapor inhalation for 6 months does not alter the expression level of GLT-1 in the FC in female CD1 mice (Alasmari et al., 2017). In the present study, daily inhalation of E-CIG vapor containing nicotine for 6 months did not affect the concentrations of glutamate in the FC. These data suggest that extracellular glutamate in the FC is transported into astrocytes mainly by GLT-1 and is then converted to glutamine. Additionally, FC receives and sends glutamate inputs from and to multiple brain regions in the mesocorticolimbic area. Thus, most of glutamate in the FC innervates other brain areas such as NAc (Britt et al., 2012). We found here that E-CIG vapor containing nicotine exposure increased the concentration of glutamate in the STR (**Figures 3C, D** and **6**). It is noteworthy that the ventral STR area receives glutamate inputs from FC, amygdala, and hippocampus (Kalivas and Volkow, 2005; Britt et al., 2012). Moreover, a prior study from our laboratory found that 6-month daily inhalation of E-CIG vapor containing nicotine downregulated striatal xCT and GLT-1, but not in the FC (Alasmari et al., 2017). The increase in glutamatergic projections to the STR (Mori et al., 1994) and the decrease of striatal glial glutamate transporters expression (Alasmari et al., 2017) may lead to high concentrations of glutamate. Elevation of glutamate concentrations in the STR may mediate the initiation and development of nicotine addiction.

Nicotine dependence has been suggested to be associated with increases in glutamine biosynthesis and concentrations in astrocytes. We demonstrate here that E-CIG exposure increases glutamine concentrations in both FC and STR (**Figures 4A–D** and **6**). These data are consistent with a prior study showing that glutamine synthesis was increased significantly in the cortex and subcortex brain regions of C57BL/6 mice injected with nicotine (2 mg/kg, s.c.) for 4 weeks (Shameem and Patel, 2012) and confirms that inhalation of nicotine induces the same alterations.

The GABAergic system has been investigated extensively as a major inhibitory neurotransmitter system strongly associated with drug dependence (Negus et al., 2000; Paterson et al., 2004; Jayaram and Steketee, 2005; Miranda et al., 2009). A prior study reported that chronic exposure to tobacco smoke (500 ml, three times/day) for 4 weeks environment or nicotine reduced the expression of GABA-B1 receptor in the hippocampus in male Sprague–Dawley rats (Li et al., 2002). Therapeutic compounds that modulate the GABA receptor have been reported to attenuate nicotine-seeking behavior in mice and rats (Fattore et al., 2002; Paterson et al., 2008; Li et al., 2017). The inhibitory role of GABA on dopamine release is suggested to be the mechanism for the attenuating effects of GABA receptors agonists on nicotine-seeking behavior (D’Souza and Markou, 2013). Although nicotine exposure (2 mg/kg, s.c.) for 4 weeks in C57BL/6 mice may increase the synthesis of GABA in the cortex (Shameem and Patel, 2012), exposure to 500 nM of nicotine for 25 min has been shown to increase GABA transmission measured by inhibitory postsynaptic currents, and this effect was followed by a long-term inhibition on the GABA neurons in the VTA (Pidoplichko et al., 2004). This indicates that chronic nicotine exposure desensitizes GABA receptors and reduces GABA transmission (Mansvelder et al., 2002). In the present study, we found that daily E-CIG vapor exposure for 6 months reduced GABA concentrations in the FC, but not in the STR (**Figures 5A–D** and **6**). The decrease of GABA level in the FC in our study suggests that persistent nicotine exposure results in further stimulation of excitatory neurotransmission in central reward areas, which may facilitate nicotine-seeking behavior.

## Conclusion

Our data indicate that chronic, daily inhalation of nicotine-containing E-CIG vapor alters the concentrations of neurotransmitters within mesocorticolimbic brain regions (**Figure 6**). The changes in neurotransmitter levels in our murine model suggest that daily, persistent use of E-CIGs may lead to addiction to nicotine. The deleterious effect of the E-CIG on the neurotransmitters may be due to nicotine exposure; however, further investigations for the effects of the vehicle used in the E-CIG on neurotransmitters’ concentrations are warranted. This raises a public health concern as it suggests that the younger generations of users, which have the highest rates of E-CIG use, might become addicted to these devices despite unknown long-term physiologic and pathologic consequences (Crotty Alexander et al., 2015). Furthermore, existing data suggest that young users of E-CIGs are more likely to start smoking conventional tobacco, which may have serious deleterious effects on human health (Leventhal et al., 2015; Goldenson et al., 2017). Further studies are needed to determine the withdrawal and relapse effects of exposure to nicotine-containing E-CIG vapor on neurotransmitter concentrations in the mesocorticolimbic brain regions.

## Data Availability

The raw data supporting the conclusions of this manuscript will be made available by the authors, without undue reservation, to any qualified researcher.

## Ethics Statement

All animal protocols were in accordance with the NIH guidelines for animal use and approved by the IACUC committee at the University of California, San Diego and San Diego VA Healthcare System.

## Author Contributions

FA participated in study design and conceptualization, drafted and revised the manuscript, performed electrochemical detection of neurotransmitters, collected the data. LCA conceptualized and designed the study, critically revised the manuscript for intellectual content, and approved the final version of the manuscript. AH performed electrochemical detection of neurotransmitters, collected the data and helped with the editing of manuscript. CB performed the animal study with electronic cigarettes containing nicotine and collected the animal brains. AM performed the animal study with electronic cigarettes containing nicotine and collected the animal brains. YS conceptualized and designed the study, critically revised the manuscript for intellectual content, and approved the final version of the manuscript.

## Conflict of Interest Statement

The authors declare that the research was conducted in the absence of any commercial or financial relationships that could be construed as a potential conflict of interest.

## Abbreviations

α-7 nAChR, alpha-7 nicotinic acetylcholine receptor; e-cigarettes, electronic cigarette; FC, frontal cortex; GABA, gamma-Aminobutyric acid; GLT-1, glutamate transporter-1; NAc, nucleus accumbens; PFC, prefrontal cortex; STR, striatum; VTA, ventral tegmental area; xCT, cystine/glutamate antiporter.

## References

[B1] Alasmari F. Crotty Alexander L. E. Nelson J. A. Schiefer I. T. Breen E. Drummond C. A. (2017). Effects of chronic inhalation of electronic cigarettes containing nicotine on glial glutamate transporters and alpha-7 nicotinic acetylcholine receptor in female CD-1 mice. Prog. Neuropsychopharmacol. Biol. Psychiatry 77, 1–8. 10.1016/j.pnpbp.2017.03.017 28347687 PMC5466499

[B2] Alasmari F. Goodwani S. McCullumsmith R. E. Sari Y. (2018). Role of glutamatergic system and mesocorticolimbic circuits in alcohol dependence. Prog. Neurobiol. 171, 32–49. 10.1016/j.pneurobio.2018.10.001 30316901 PMC6261463

[B3] Awtry T. L. Werling L. L. (2003). Acute and chronic effects of nicotine on serotonin uptake in prefrontal cortex and hippocampus of rats. Synapse 50, 206–211. 10.1002/syn.10259 14515338

[B4] Britt J. P. Benaliouad F. McDevitt R. A. Stuber G. D. Wise R. A. Bonci A. (2012). Synaptic and behavioral profile of multiple glutamatergic inputs to the nucleus accumbens. Neuron 76, 790–803. 10.1016/j.neuron.2012.09.040 23177963 PMC3607383

[B5] Caillé S. Parsons L. H. (2004). Intravenous heroin self-administration decreases GABA efflux in the ventral pallidum: an *in vivo* microdialysis study in rats. Eur. J. Neurosci. 20, 593–596. 10.1111/j.1460-9568.2004.03497.x 15233770

[B6] Canistro D. Vivarelli F. Cirillo S. Marquillas C. B. Buschini A. Lazzaretti M. (2017). E-cigarettes induce toxicological effects that can raise the cancer risk. Sci. Rep. 7, 2028. 10.1038/s41598-017-02317-8 28515485 PMC5435699

[B7] Carr D. B. Sesack S. R. (2000). GABA-containing neurons in the rat ventral tegmental area project to the prefrontal cortex. Synapse 38, 114–123. 10.1002/1098-2396(200011)38:2<114::AID-SYN2>3.0.CO;2-R 11018785

[B8] Cheng Q. Yakel J. L. (2014). Presynaptic alpha7 nicotinic acetylcholine receptors enhance hippocampal mossy fiber glutamatergic transmission *via* PKA activation. J. Neurosci. 34, 124–133. 10.1523/JNEUROSCI.2973-13.2014 24381273 PMC3866480

[B9] Corrigall W. A. Coen K. M. (1991). Selective dopamine antagonists reduce nicotine self-administration. Psychopharmacology 104, 171–176. 10.1007/BF02244174 1876661

[B10] Crotty Alexander L. E. Drummond C. A. Hepokoski M. Mathew D. P. Moshensky A. Willeford A. (2018). Chronic inhalation of E-cigarette vapor containing nicotine disrupts airway barrier function and induces systemic inflammation and multi-organ fibrosis in mice. Am. J. Physiol. Regul. Integr. Comp. Physiol. 314, R834–R847. 10.1152/ajpregu.00270.2017 29384700 PMC6032308

[B11] Crotty Alexander L. E. Vyas A. Schraufnagel D. E. Malhotra A. (2015). Electronic cigarettes: the new face of nicotine delivery and addiction. J. Thorac. Dis. 7, E248–E251. 10.3978/j.issn.2072-1439.2015.07.37 26380791 PMC4561260

[B12] D’Souza M. S. Markou A. (2013). The “stop” and “go” of nicotine dependence: role of GABA and glutamate. Cold Spring Harbor Perspect. Med. 3, a012146. 10.1101/cshperspect.a012146 PMC3662348 23732855

[B13] Das S. C. Althobaiti Y. S. Alshehri F. S. Sari Y. (2016). Binge ethanol withdrawal: effects on post-withdrawal ethanol intake, glutamate–glutamine cycle and monoamine tissue content in P rat model. Behav. Brain Res. 303, 120–125. 10.1016/j.bbr.2016.01.052 26821293 PMC4779422

[B14] Das S. C. Yamamoto B. K. Hristov A. M. Sari Y. (2015). Ceftriaxone attenuates ethanol drinking and restores extracellular glutamate concentration through normalization of GLT-1 in nucleus accumbens of male alcohol-preferring rats. Neuropharmacology 97, 67–74. 10.1016/j.neuropharm.2015.05.009 26002627 PMC4537362

[B15] Dawkins L. Turner J. Hasna S. Soar K. (2012). The electronic-cigarette: effects on desire to smoke, withdrawal symptoms and cognition. Addict. Behav. 37, 970–973. 10.1016/j.addbeh.2012.03.004 22503574

[B16] Deehan G. A. Hauser S. R. Waeiss R. A. Knight C. P. Toalston J. E. Truitt W. A. (2015). Co-administration of ethanol and nicotine: the enduring alterations in the rewarding properties of nicotine and glutamate activity within the mesocorticolimbic system of female alcohol-preferring (P) rats. Psychopharmacology 232, 4293–4302. 10.1007/s00213-015-4056-1 26306917 PMC4899841

[B17] Delnevo C. D. Giovenco D. P. Steinberg M. B. Villanti A. C. Pearson J. L. Niaura R. S. (2015). Patterns of electronic cigarette use among adults in the United States. Nicotine Tob. Res. 18, 715–719. 10.1093/ntr/ntv237 26525063 PMC5896829

[B18] Dewey S. L. Brodie J. D. Gerasimov M. Horan B. Gardner E. L. Ashby C. R. (1999). A pharmacologic strategy for the treatment of nicotine addiction. Synapse 31, 76–86. 10.1002/(SICI)1098-2396(199901)31:1<76::AID-SYN10>3.0.CO;2-Y 10025686

[B19] DuBois D. W. Damborsky J. C. Fincher A. S. Frye G. D. Winzer-Serhan U. H. (2013). Varenicline and nicotine enhance GABAergic synaptic transmission in rat CA1 hippocampal and medial septum/diagonal band neurons. Life Sci. 92, 337–344. 10.1016/j.lfs.2012.12.013 23352971 PMC3598584

[B20] Edwards N. J. Tejeda H. A. Pignatelli M. Zhang S. McDevitt R. A. Wu J. (2017). Circuit specificity in the inhibitory architecture of the VTA regulates cocaine-induced behavior. Nat. Neurosci. 20, 438–448. 10.1038/nn.4482 28114294

[B21] Etter J.-F. Eissenberg T. (2015). Dependence levels in users of electronic cigarettes, nicotine gums and tobacco cigarettes. Drug Alcohol Depend. 147, 68–75. 10.1016/j.drugalcdep.2014.12.007 25561385 PMC4920051

[B22] Fattore L. Cossu G. Martellotta M. C. Fratta W. (2002). Baclofen antagonizes intravenous self-administration of nicotine in mice and rats. Alcohol Alcohol. 37, 495–498. 10.1093/alcalc/37.5.495 12217945

[B23] Feduccia A. A. Chatterjee S. Bartlett S. E. (2012). Neuronal nicotinic acetylcholine receptors: neuroplastic changes underlying alcohol and nicotine addictions. Front. Mol. Neurosci. 5, 83. 10.3389/fnmol.2012.00083 22876217 PMC3411089

[B24] Foulds J. Veldheer S. Yingst J. Hrabovsky S. Wilson S. J. Nichols T. T. (2014). Development of a questionnaire for assessing dependence on electronic cigarettes among a large sample of ex-smoking E-cigarette users. Nicotine Tob. Res. 17, 186–192. 10.1093/ntr/ntu204 25332459 PMC4838001

[B25] Fuxe K. Jansson A. Jansson A. Andersson K. Eneroth P. Agnati L. (1990). Chronic nicotine treatment increases dopamine levels and reduces dopamine utilization in substantia nigra and in surviving forebrain dopamine nerve terminal systems after a partial di-mesencephalic hemitransection. Naunyn Schmiedebergs Arch. Pharmacol. 341, 171–181. 10.1007/BF00169727 2342600

[B26] Goldenson N. I. Leventhal A. M. Stone M. D. McConnell R. S. Barrington-Trimis J. L. (2017). Associations of electronic cigarette nicotine concentration with subsequent cigarette smoking and vaping levels in adolescents. JAMA Pediatr. 171, 1192–1199. 10.1001/jamapediatrics.2017.3209 29059261 PMC5779618

[B27] Goodwani S. Saternos H. Alasmari F. Sari Y. (2017). Metabotropic and ionotropic glutamate receptors as potential targets for the treatment of alcohol use disorder. Neurosci. Biobehav. Rev. 77, 14–31. 10.1016/j.neubiorev.2017.02.024 28242339 PMC5446930

[B28] Han F. Shioda N. Moriguchi S. Qin Z.-H. Fukunaga K. (2008). Downregulation of glutamate transporters is associated with elevation in extracellular glutamate concentration following rat microsphere embolism. Neurosci. Lett. 430, 275–280. 10.1016/j.neulet.2007.11.021 18079058

[B29] Hwang J. H. Lyes M. Sladewski K. Enany S. McEachern E. Mathew D. P. (2016). Electronic cigarette inhalation alters innate immunity and airway cytokines while increasing the virulence of colonizing bacteria. J. Mol. Med., 94(6), 667–679. 10.1007/s00109-016-1378-3 26804311

[B30] Ishikawa H. Ohtsuki T. Ishiguro H. Yamakawa-Kobayashi K. Endo K. Lin Y.-L. (1999). Association between serotonin transporter gene polymorphism and smoking among Japanese males. Cancer Epidemiol. Prev. Biomarkers 8, 831–833. 10498403

[B31] Jayaram P. Steketee J. D. (2005). Effects of cocaine-induced behavioural sensitization on GABA transmission within rat medial prefrontal cortex. Eur. J. Neurosci. 21, 2035–2039. 10.1111/j.1460-9568.2005.04000.x 15869498

[B32] Kalivas P. W. Volkow N. D. (2005). The neural basis of addiction: a pathology of motivation and choice. Am. J. Psychiatry 162, 1403–1413. 10.1176/appi.ajp.162.8.1403 16055761

[B33] Knackstedt L. A. LaRowe S. Mardikian P. Malcolm R. Upadhyaya H. Hedden S. (2009). The role of cystine-glutamate exchange in nicotine dependence in rats and humans. Biol. Psychiatry 65, 841–845. 10.1016/j.biopsych.2008.10.040 19103434 PMC2756612

[B34] Konradsson-Geuken Å. Gash C. R. Alexander K. Pomerleau F. Huettl P. Gerhardt G. A. (2009). Second-by-second analysis of alpha 7 nicotine receptor regulation of glutamate release in the prefrontal cortex of awake rats. Synapse 63, 1069–1082. 10.1002/syn.20693 19637277 PMC2759414

[B35] LaLumiere R. T. Kalivas P. W. (2008). Glutamate release in the nucleus accumbens core is necessary for heroin seeking. J. Neurosci. 28, 3170–3177. 10.1523/JNEUROSCI.5129-07.2008 18354020 PMC6670700

[B36] Leventhal A. M. Strong D. R. Kirkpatrick M. G. Unger J. B. Sussman S. Riggs N. R. (2015). Association of electronic cigarette use with initiation of combustible tobacco product smoking in early adolescence. JAMA 314, 700–707. 10.1001/jama.2015.8950 26284721 PMC4771179

[B37] Li S. P. Park M. S. Bahk J. Y. Kim M. O. (2002). Chronic nicotine and smoking exposure decreases GABA B1 receptor expression in the rat hippocampus. Neurosci. Lett. 334, 135–139. 10.1016/S0304-3940(02)01065-0 12435490

[B38] Li X. Sturchler E. Kaczanowska K. Cameron M. Finn M. Griffin P. (2017). KK-92A, a novel GABA B receptor positive allosteric modulator, attenuates nicotine self-administration and cue-induced nicotine seeking in rats. Psychopharmacology 234, 1633–1644. 10.1007/s00213-017-4594-9 28382542

[B39] Mansvelder H. D. Keath J. R. McGehee D. S. (2002). Synaptic mechanisms underlie nicotine-induced excitability of brain reward areas. Neuron 33, 905–919. 10.1016/S0896-6273(02)00625-6 11906697

[B40] Margham J. McAdam K. Forster M. Liu C. Wright C. Mariner D. (2016). Chemical composition of aerosol from an E-cigarette: a quantitative comparison with cigarette smoke. Chem. Res. Toxicol. 29, 1662–1678. 10.1021/acs.chemrestox.6b00188 27641760

[B41] Mifsud J.-C. Hernandez L. Hoebel B. G. (1989). Nicotine infused into the nucleus accumbens increases synaptic dopamine as measured by *in vivo* microdialysis. Brain Res. 478, 365–367. 10.1016/0006-8993(89)91518-7 2924134

[B42] Miranda F. Jiménez J. C. Cedillo L. N. Sandoval-Sánchez A. Millán-Mejía P. Sánchez-Castillo H. (2009). The GABA-B antagonist 2-hydroxysaclofen reverses the effects of baclofen on the discriminative stimulus effects of D-amphetamine in the conditioned taste aversion procedure. Pharmacol. Biochem. Behav. 93, 25–30. 10.1016/j.pbb.2009.04.002 19361543

[B43] Mori A. Takahashi T. Miyashita Y. Kasai H. (1994). Two distinct glutamatergic synaptic inputs to striatal medium spiny neurones of neonatal rats and paired-pulse depression. J. Physiol. 476, 217–228. 10.1113/jphysiol.1994.sp020125 8046639 PMC1160435

[B44] Munafo M. R. Roberts K. Johnstone E. C. Walton R. T. Yudkin P. L. (2005). Association of serotonin transporter gene polymorphism with nicotine dependence: no evidence for an interaction with trait neuroticism. Pers. Individ. Dif. 38, 843–850. 10.1016/j.paid.2004.06.008

[B45] Nakamura K. (2013). The role of the dorsal raphé nucleus in reward-seeking behavior. Front. Integr. Neurosci. 7 (60), 1–18. 10.3389/fnint.2013.00060 23986662 PMC3753458

[B46] Negus S. S. Mello N. K. Fivel P. A. (2000). Effects of GABA agonists and GABA-A receptor modulators on cocaine discrimination in rhesus monkeys. Psychopharmacology 152, 398–407. 10.1007/s002130000543 11140332

[B47] Palipudi K. M. Group G. C. Mbulo L. Group G. C. Morton J. Group G. C. (2015). Awareness and current use of electronic cigarettes in Indonesia, Malaysia, Qatar, and Greece: findings from 2011–2013 Global adult tobacco surveys. Nicotine Tob. Res. 18, 501–507. 10.1093/ntr/ntv081 25895951 PMC5100820

[B48] Paterson N. E. Froestl W. Markou A. (2004). The GABAB receptor agonists baclofen and CGP44532 decreased nicotine self-administration in the rat. Psychopharmacology 172, 179–186. 10.1007/s00213-003-1637-1 14610636

[B49] Paterson N. E. Froestl W. Markou A. (2005). Repeated administration of the GABAB receptor agonist CGP44532 decreased nicotine self-administration, and acute administration decreased cue-induced reinstatement of nicotine-seeking in rats. Neuropsychopharmacology 30, 119. 10.1038/sj.npp.1300524 15266350

[B50] Paterson N. E. Vlachou S. Guery S. Kaupmann K. Froestl W. Markou A. (2008). Positive modulation of GABAB receptors decreased nicotine self-administration and counteracted nicotine-induced enhancement of brain reward function in rats. J. Pharmacol. Exp. Ther. 326, 306–314. 10.1124/jpet.108.139204 18445779 PMC2574924

[B51] Paxinos G. Franklin K. B. (2004) The Mouse Brain in Stereotaxic Coordinates. Elsevier Academic Press (USA).

[B52] Perez X. A. Ly J. McIntosh J. M. Quik M. (2012). Long-term nicotine exposure depresses dopamine release in nonhuman primate nucleus accumbens. J. Pharmacol. Exp. Ther. 342, 335–344. 10.1124/jpet.112.194084 22562772 PMC3400796

[B53] Pidoplichko V. I. Noguchi J. Areola O. O. Liang Y. Peterson J. Zhang T. (2004). Nicotinic cholinergic synaptic mechanisms in the ventral tegmental area contribute to nicotine addiction. Learn. Memory 11, 60–69. 10.1101/lm.70004 PMC321315 14747518

[B54] Pistillo F. Clementi F. Zoli M. Gotti C. (2015). Nicotinic, glutamatergic and dopaminergic synaptic transmission and plasticity in the mesocorticolimbic system: focus on nicotine effects. Prog. Neurobiol. 124, 1–27. 10.1016/j.pneurobio.2014.10.002 25447802

[B55] Polosa R. Benowitz N. L. (2011). Treatment of nicotine addiction: present therapeutic options and pipeline developments. Trends Pharmacol. Sci. 32, 281–289. 10.1016/j.tips.2010.12.008 21256603 PMC5564372

[B56] Rademacher L. Prinz S. Winz O. Henkel K. Dietrich C. A. Schmaljohann J. (2016). Effects of smoking cessation on presynaptic dopamine function of addicted male smokers. Biol. Psychiatry 80, 198–206. 10.1016/j.biopsych.2015.11.009 26803340

[B57] Ribeiro E. B. Bettiker R. L. Bogdanov M. Wurtman R. J. (1993). Effects of systemic nicotine on serotonin release in rat brain. Brain Res. 621, 311–318. 10.1016/0006-8993(93)90121-3 8242344

[B58] Sari Y. Toalston J. E. Rao P. S. Bell R. L. (2016). Effects of ceftriaxone on ethanol, nicotine or sucrose intake by alcohol-preferring (P) rats and its association with GLT-1 expression. Neuroscience 326, 117–125. 10.1016/j.neuroscience.2016.04.004 27060486 PMC4853280

[B59] Saunders A. Oldenburg I. A. Berezovskii V. K. Johnson C. A. Kingery N. D. Elliott H. L. (2015). A direct GABAergic output from the basal ganglia to frontal cortex. Nature 521, 85. 10.1038/nature14179 25739505 PMC4425585

[B60] Scherma M. Justinová Z. Zanettini C. Panlilio L. V. Mascia P. Fadda P. (2012). The anandamide transport inhibitor AM404 reduces the rewarding effects of nicotine and nicotine induced dopamine elevations in the nucleus accumbens shell in rats. Br. J. Pharmacol. 165, 2539–2548. 10.1111/j.1476-5381.2011.01467.x 21557729 PMC3423245

[B61] Semba J. Wakuta M. (2008). Chronic effect of nicotine on serotonin transporter mRNA in the raphe nucleus of rats: reversal by co-administration of bupropion. Psychiatry Clin. Neurosci. 62, 435–441. 10.1111/j.1440-1819.2008.01822.x 18778441

[B62] Shameem M. Patel A. B. (2012). Glutamatergic and GABAergic metabolism in mouse brain under chronic nicotine exposure: implications for addiction. PLoS One 7, e41824. 10.1371/journal.pone.0041824 22848621 PMC3405019

[B63] Singh T. (2016). Tobacco use among middle and high school students—United States, 2011–2015. MMWR Morbidity and mortality weekly report. 65. 10.15585/mmwr.mm6514a1 27077789

[B64] Smolka M. N. Reimold M. Kobiella A. Reischl G. Rietschel M. Heinz A. (2019). Smoking moderates association of 5-HTTLPR and in vivo availability of serotonin transporters. Eur. Neuropsychopharmacol. 29, 171–178. 10.1016/j.euroneuro.2018.08.509 30587400

[B65] Subramaniyan M. Dani J. A. (2015). Dopaminergic and cholinergic learning mechanisms in nicotine addiction. Ann. N. Y. Acad. Sci. 1349, 46–63. 10.1111/nyas.12871 26301866 PMC4564314

[B66] Takahashi H. Takada Y. Nagai N. Urano T. Takada A. (1998). Nicotine increases stress-induced serotonin release by stimulating nicotinic acetylcholine receptor in rat striatum. Synapse 28, 212–219. 10.1002/(SICI)1098-2396(199803)28:3<212::AID-SYN4>3.0.CO;2-D 9488506

[B67] Tizabi Y. Bai L. Copeland R. L. Jr. Taylor R. E. (2007). Combined effects of systemic alcohol and nicotine on dopamine release in the nucleus accumbens shell. Alcohol Alcohol. 42, 413–416. 10.1093/alcalc/agm057 17686828

[B68] Tizabi Y. Copeland R. L. Louis V. A. Taylor R. E. (2002). Effects of combined systemic alcohol and central nicotine administration into ventral tegmental area on dopamine release in the nucleus accumbens. Alcohol. Clin. Exp. Res. 26, 394–399. 10.1111/j.1530-0277.2002.tb02551.x 11923594

[B69] Vardavas C. I. Anagnostopoulos N. Kougias M. Evangelopoulou V. Connolly G. N. Behrakis P. K. (2012). Short-term pulmonary effects of using an electronic cigarette: impact on respiratory flow resistance, impedance, and exhaled nitric oxide. Chest J. 141, 1400–1406. 10.1378/chest.11-2443 22194587

[B70] Wang B. W. Liao W. N. Chang C. T. Wang S. J. (2006). Facilitation of glutamate release by nicotine involves the activation of a Ca2+/calmodulin signaling pathway in rat prefrontal cortex nerve terminals. Synapse 59, 491–501. 10.1002/syn.20267 16565963

[B71] Watanabe M. A. E. Nunes S. O. V. Amarante M. K. Guembarovski R. L. Oda J. M. M. De Lima K. W. A. (2011). Genetic polymorphism of serotonin transporter 5-HTTLPR: involvement in smoking behaviour. J. Genet. 90, 179–185. 10.1007/s12041-011-0037-2 21677407

[B72] Yan Y. Peng C. Arvin M. C. Jin X.-T. Kim V. J. Ramsey M. D. (2018). Nicotinic cholinergic receptors in VTA glutamate neurons modulate excitatory transmission. Cell Rep. 23, 2236–2244. 10.1016/j.celrep.2018.04.062 29791835 PMC5999341

[B73] Yu V. Rahimy M. Korrapati A. Xuan Y. Zou A. E. Krishnan A. R. (2016). Electronic cigarettes induce DNA strand breaks and cell death independently of nicotine in cell lines. Oral Oncol. 52, 58–65. 10.1016/j.oraloncology.2015.10.018 26547127 PMC4891196

[B74] Zhong J. Cao S. Gong W. Fei F. Wang M. (2016). Electronic cigarettes use and intention to cigarette smoking among never-smoking adolescents and young adults: a meta-analysis. Int. J. Environ. Res. Public Health 13, 465. 10.3390/ijerph13050465 PMC4881090 27153077

